# The teaching swimming for understanding framework: an integrated approach to aquatic education for children and instructors

**DOI:** 10.3389/fspor.2026.1916430

**Published:** 2026-08-12

**Authors:** Gabriele Signorini, Raffaele Scurati, Roberto Del Bianco, Pietro Luigi Invernizzi, Marta Rigon

**Affiliations:** 1 Department of Biomedical Sciences for Health, Università Degli Studi di Milano, Milan, Italy; 2 Federazione Italiana Nuoto, Roma, Italy

**Keywords:** acquisition, non-linear pedagogy, retention, teaching games for understanding, training course, transfer

## Abstract

This study evaluates the impact of the Teaching Swimming for Understanding (TSfU) program within a comprehensive aquatic education framework that integrates motor, cognitive, emotional, and social learning, extending beyond sports success. A total of 200 children aged 5–8 years attending swimming lessons and 44 swimming instructors with federal licenses were recruited for the study. The instructors were divided into an experimental group that participated in a training course oriented to TSfU principles, while the control group maintained their federal formation. Children were divided into the same groups depending on their swimming instructors. Swimming lessons for both groups were recorded and analyzed to assess didactic capacity, methodological capacity, and the amount and type of physical activity among children. Moreover, questionnaires were provided to instructors to assess empathy and self-control. Children's actual and perceived water competence were assessed using two pictorial scales. All measures were administered before and after the instructor's training course and after one month of rest to assess retention. Finally, the parents of involved children were interviewed after the retention phase with semi-structured interviews to assess the transfer of swimming skills to environments outside the swimming pool (i.e., aquatic competence in open water). The TSfU group showed improvement in learners' aquatic competences, as well as perceived aquatic competencies. Meanwhile, instructors who participated in TSfU training showed notable improvements in didactic capacity values (in particular communication, organizational, and motivational skills), the number of teaching styles used, the amount of activity provided, and interaction with learners. These findings align with constructivism and the ecological-dynamics approach, supporting active, personalized, and reflective teaching. The constructivist and ecological dynamics methods encouraged retention and real-world transfer of aquatic skills, particularly in water safety, environmental awareness, and self-awareness. Instructors benefited from professional development in pedagogical and interpersonal skills, supporting the approach's sustainability. Overall, the study highlights the effectiveness of personalized, dynamic, multi-teaching strategies in fostering enduring learning outcomes and provides valuable insights for future research and practical applications in aquatic education.

## 1 Introduction

Traditional swimming instruction is commonly organized around the progressive reproduction of predefined technical movements. In this approach, instructors typically demonstrate a swimming skill, provide detailed technical instructions, and ask learners to repeat the movement until an expected performance standard is achieved. Although this method can support the acquisition of specific swimming techniques, its predominantly linear structure may provide limited opportunities for autonomy, exploration, decision-making, and adaptation to individual learning needs (1–3). These limitations are particularly relevant in aquatic education, where competence involves more than the reproduction of formal swimming strokes. Aquatic competence includes a broad range of psychomotor, cognitive, and affective capacities that enable individuals to perceive environmental information, regulate their actions in relation to water conditions, solve movement problems, evaluate risks, and behave appropriately in unfamiliar aquatic situations (4, 5). Accordingly, swimming skill represents an important component of aquatic competence but should not be treated as equivalent to the broader capacity to act safely and effectively in, on, and around water.

The pedagogical foundation of the present intervention is Teaching Games for Understanding (TGfU), originally developed by Bunker and Thorpe as an alternative to technique-dominated approaches to sports instruction (6). In TGfU, learners should first engage with meaningful and modified situations and subsequently develop the technical skills required to solve the problems emerging from those situations. Rather than treating technique as the exclusive starting point of instruction, the model emphasizes understanding, decision-making, problem-solving, contextual awareness, and the active participation of the learner (7, 8).

Although TGfU was initially conceived for games and team sports, its central principles are relevant to aquatic education. Learning to act effectively in water requires the continuous integration of perception, movement, decision-making, and adaptation. Aquatic competence therefore cannot be reduced to the execution of isolated or closed technical skills. Children must learn to recognize the opportunities and constraints offered by the aquatic environment, select appropriate actions, manage uncertainty, and modify their behavior in response to changing task and environmental conditions (3). This perspective is consistent with nonlinear pedagogy, which conceptualizes motor learning as an adaptive process emerging from interactions among the learner, the task, and the environment (9–11). From this standpoint, learning activities should not prescribe a single movement solution in every situation but should create conditions in which learners can explore and stabilize different functional responses. The manipulation of task, environmental, and individual constraints may guide this exploration and facilitate the discovery of effective aquatic behaviors (12–14).

The constraints used in aquatic education may include changes in depth, available support, body orientation, distance, direction, object use, water stability, visibility, or the informational characteristics of the environment. Their pedagogical value does not lie in introducing variability for its own sake, but in helping learners establish functional relations between perceived information and movement. The suitability of each constraint must therefore be evaluated in relation to the learner's characteristics, the intended educational objective, and the safety requirements of the task. Representative learning design provides an additional basis for structuring these learning experiences. It proposes that practice activities should preserve the perceptual information and action possibilities that characterize the situations in which the acquired competencies will subsequently be used (15). In aquatic education, this means that transfer is more likely when children experience meaningful variations that require them to perceive changing conditions, select an appropriate response, and adapt their movements accordingly, rather than practising exclusively under highly stable and predictable conditions (4).

Based on these principles, Teaching Swimming for Understanding (TSfU) was developed as an aquatic adaptation of TGfU (3). The purpose of TSfU is not to transfer the TGfU model mechanically from games to swimming, but to reinterpret its main pedagogical principles in relation to the specific perceptual, motor, emotional, and safety demands of aquatic environments.

Three principal adaptations were required. First, the game-based problems used in TGfU were replaced by meaningful aquatic challenges. These challenges involve goals such as maintaining flotation, controlling breathing, changing body position, travelling through water, entering and exiting safely, interacting with objects, and responding to variations in depth, support, stability, or direction. Technical swimming skills are introduced as possible solutions to these problems rather than solely as isolated movements to be reproduced from the beginning of the learning process. Second, the tactical-understanding component of TGfU was reformulated as aquatic understanding. In TSfU, understanding refers to the learner's ability to interpret the relationship among the body, the water, the task, and the surrounding environment. Children are encouraged to recognize why a particular action is effective, how water conditions influence movement, and how a solution may need to be modified when the context changes. Third, the teacher-led questioning strategy characteristic of TGfU was adapted to aquatic instruction. Instructors use questions, guided exploration, and short periods of reflection to help children identify movement problems and evaluate possible solutions. Questions are intended to direct attention toward relevant information rather than to replace practical experience or provide children with predetermined verbal answers.

Direct instruction nevertheless remains necessary when safety-critical skills are taught, when the risks associated with an incorrect response are substantial, or when a learner requires explicit assistance. Examples include head-first entries, unexpected falls into water, jumps, emergency behaviors, and procedures for assisting another person. Even in these circumstances, children can be encouraged to identify risks and reflect on safe options before or after the instructor provides explicit technical guidance. TSfU should therefore be understood as a flexible integration of guided discovery and direct instruction rather than as the elimination of instructor-led teaching. The resulting TSfU model maintains the understanding-oriented structure of TGfU while incorporating the distinctive demands of aquatic learning. It combines structured learning objectives with task variability, guided discovery, active reflection, external attentional focus, and progressive modifications of the environment (3, 16). For example, the level of support provided during an aquatic task can be gradually reduced by moving from a stable pool edge to ropes or other less stable supports. Such modifications allow children to explore balance, propulsion, and flotation while progressively increasing their independence.

Within the TSfU framework, learning is organized around acquisition, retention, and transfer. Acquisition concerns the development of functional aquatic competencies through active experience, practice, and problem-solving. Retention concerns the stabilization and availability of these competencies following a period without structured practice. Meaningful participation, perceived competence, intrinsic motivation, and the broader development of physical literacy may support continued engagement and contribute to the maintenance of learning over time (17–19). These factors should be regarded as theoretically plausible contributors to retention unless their mediating role is directly assessed. Transfer concerns the ability to apply competencies acquired during instruction to different activities, tasks, and aquatic environments. This dimension is especially important for water safety because competence demonstrated in a controlled swimming pool does not necessarily guarantee an effective response in the sea, a lake, a river, or an unexpected open-water situation (4, 5). Differences in temperature, clothing, waves, currents, visibility, depth, emotional pressure, and available support may substantially modify the demands placed on the learner.

The role of the instructor is central to the implementation of this approach. Moving from predominantly technique-led instruction to TSfU requires instructors to design appropriate aquatic problems, manipulate task constraints, observe individual responses, use questioning effectively, provide relevant feedback, and balance learner autonomy with safety. In the present study, the Knowledge, Skills, Abilities, and other characteristics framework was used to organize and assess these instructor competencies (3). In particular, KSAO frameworks (Supplementary Table S1) originate in job and work analysis, provide a specific structured means of distinguishing acquired knowledge, observable skills, broader abilities, and other personal or professional characteristics relevant to effective performance (20, 21).

**Figure 1:**
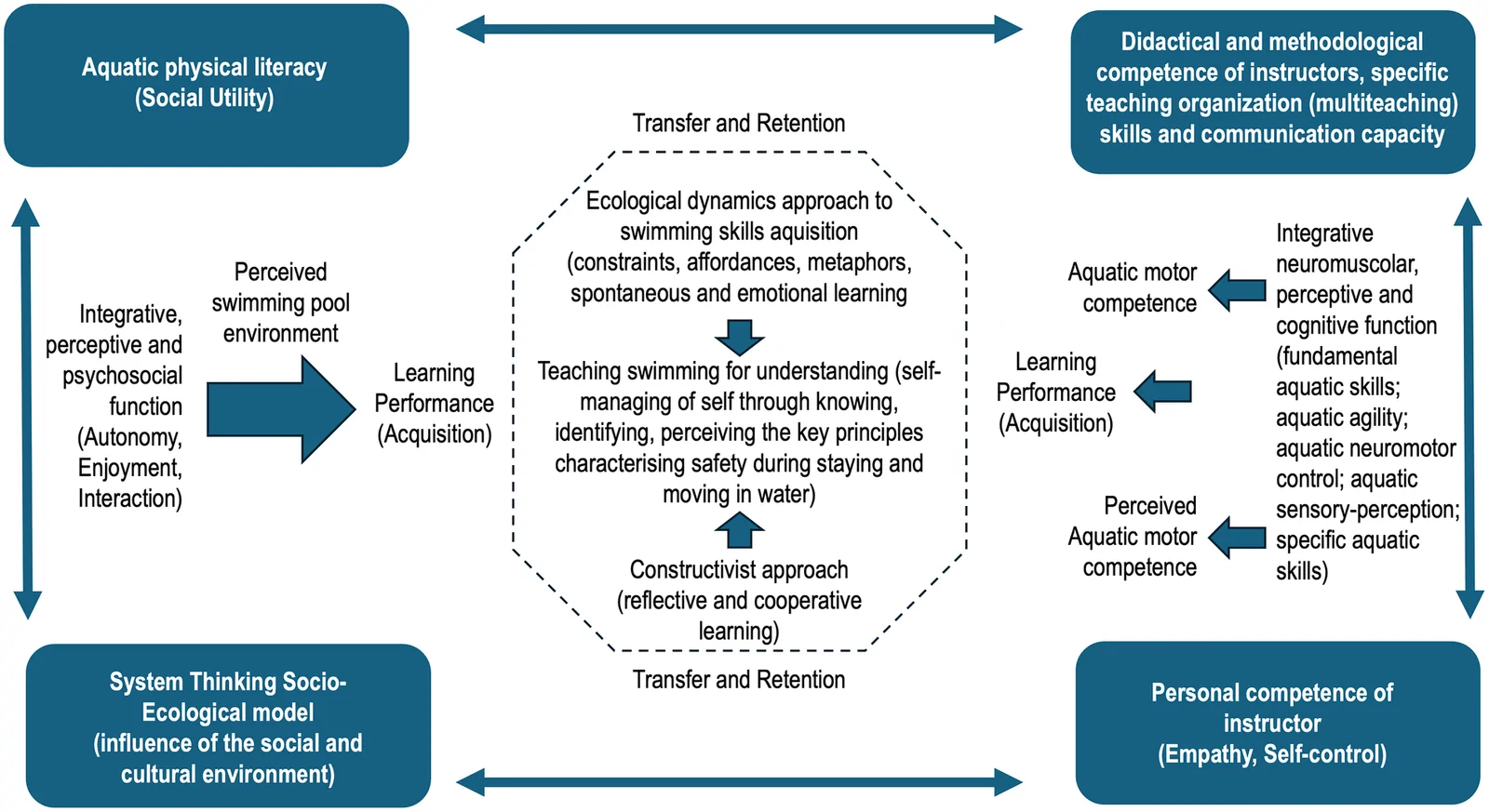
Framework TSfU: a new paradigm for teaching swimming.

Figure 1 summarizes the elements that characterize the framework, highlighting the parallels between the teacher training process and the educational process for children.

The relationships among the learner, the task, and the environment represented in the figure can also be interpreted in connection with ecological dynamics and nonlinear pedagogy (10) while active problem-solving and meaning-making are consistent with the constructivist principles underlying TGfU (8). The framework also connects children's actual and perceived aquatic competence and physical literacy with instructors’ didactic-methodological and personal competencies. This multidimensional perspective recognizes that aquatic learning involves motor, cognitive, affective, and social outcomes and is influenced by the broader educational context (22–24). Overall, the figure places TGfU and its aquatic adaptation, TSfU, at the center of an adaptive and learner-centered educational process aimed at developing meaningful, stable, and transferable aquatic competencies.

Despite increasing interest in learner-centered and nonlinear approaches to motor education, swimming instruction continues to be predominantly technique-oriented, and evidence regarding understanding-oriented aquatic interventions remains limited (2, 25, 26). In particular, few studies have examined whether a sustained TSfU intervention can simultaneously improve instructors’ pedagogical competencies and children's aquatic outcomes, or whether the competencies developed through this approach are retained and transferred beyond the original instructional environment (3).

The study therefore investigated the effectiveness of a 2-year TSfU intervention implemented in a multifunctional aquatic center, compared with a traditional technique-based swimming instruction model. It examined changes in instructors’ KSAO and children's psychomotor, cognitive, and psychosocial outcomes. It also assessed the retention of the children's outcomes following a one-month period without practice and the reported transfer of aquatic competencies and behaviors to different aquatic environments. Accordingly, the following research questions were formulated:

1. To what extent does a two-year TSfU intervention improve instructors’ knowledge, skills, abilities, and other relevant characteristics compared with traditional technique-based swimming instruction, and to what extent do changes in instructor competencies occur alongside improvements in children's psychomotor, cognitive, and psychosocial outcomes?
2. To what extent are the aquatic motor, cognitive, and psychosocial outcomes developed through TSfU retained following a one-month period without practice, compared with the outcomes of traditional swimming instruction?
3. To what extent does TSfU facilitate the reported transfer of acquired aquatic competencies and behaviors from the swimming pool to different aquatic environments, including the sea, lakes, and rivers, compared with a traditional technique-based approach?

## 2 Materials and methods

### 2.1 Participants

The participants in the study are swimming instructors from a large sports club in the hinterland of Milan, as well as the children enrolled in the swimming courses they teach. A total of 200 children aged 5–8 years attending swimming lessons were recruited for the study. The sample size was determined using G*Power (version 3.1.9.4) with 95% power and an estimated effect size of rho = 0.3. For this calculation, the point-biserial correlation (*t*-test, one-tailed, alpha = 0.05) was used. To meet the required statistical power, 111 participants were needed. Inclusion criteria included enrollment in the introductory swimming course and possession of the necessary medical certificate confirming fitness for sports. The exclusion criterion was a lack of consent to participate in the study and to have data processed.

Additionally, 44 swimming instructors with federal licenses voluntarily participated in the study. The statistical power for this sample size was calculated to be 95%. Since the primary measures for swimming instructors were analyzed using two-way ANOVA, the statistical power was evaluated using the *F*-test “ANOVA: repeated measures, within factors,” with an effect size *f* = 0.25, an alpha value of 0.05, one group, and three repeated measures (which would represent the minimum number of repeated measures in the analysis). Instructors and their respective children were randomly assigned (using randomization software, www.random.org) to either an experimental group using the TSfU methodology or a control group using the traditional swimming methodology (TS). Anthropometric data for both groups are presented in Table 1.

**Table 1:** Participant demographics.

	First year		Second year	
	TSfU	TS	TSfU	TS
Children				
Participants (n)	110	90	100	90
Males (n)	64	42	60	42
Females (n)	46	48	40	48
Age (years)	5.9 ± 1.7	6.2 ± 1.6	7.9 ± 1.7	8.2 ± 1.6
Height (cm)	115.4 ± 11.8	117.9 ± 12.1	118.3 ± 10.6	120.1 ± 11.3
Weight (kg)	20.8 ± 4.5	20.8 ± 4.7	23.9 ± 2.3	22.5 ± 4.2
BMI (kg/m^2^)	15.5 ± 1.6	14.8 ± 1.7	17.3 ± 2.1	16.8 ± 1.9
Instructors				
Participants (n)	22	22	22	22
Males (n)	12	11	12	11
Females (n)	10	11	10	11
Age (years)	30.4 ± 7.4	32.3 ± 9.8	31.4 ± 7.4	33.3 ± 9.8
Teaching experience (years)	4.3 ± 4.1	5.8 ± 2.8	5.3 ± 4.1	6.8 ± 2.8

TSfU, experimental group (teaching swimming for understanding); TS, control group (traditional swimming).

The TSfU instructors participated in a 16-h training course of four 4-h sessions. Children attended one swimming lesson per week for 36 weeks each year, with each lesson lasting 45 min. Any dropouts during the two-year intervention are reported in Table 1.

Before participation, all instructors, participants and the parents or legal guardians of the children received a detailed explanation of the study procedures, potential risks, and expected benefits. Written informed consent was obtained from all teacher participants and from the parents or legal guardians of the children involved in the study. Participants were informed that they could withdraw from the study at any time without penalty or the need to provide a reason. All personal data were anonymized prior to analysis to ensure confidentiality. The study was conducted in accordance with the principles of the Declaration of Helsinki and was approved by the Ethics Committee of the University of Milan (protocol no. 39/25).

### 2.2 Measures

Several tools were used to measure the effects of interventions. The researchers involved in data collection limited themselves to observing the lessons, without participating in the teaching process, and were unaware of the group assignments (TSfU and TS).

The Internship Evaluation Sheet in Physical Education and Sports (IESPES) is an instrument designed to measure the different teaching knowledge acquired through real practice in the field, highlighted in the application context of the swimming lessons. The main areas evaluated were the instructor's communication, didactic organization, linear teaching, and motivation capacity. Each item was evaluated using a descriptor schedule and could be scored from 0 to 5. Scores higher than 3 are considered a fair level of didactical competence, as competence scores 4 and 5 are higher than the neutral descriptor (3 points) (3, 27–29).

The Instrument for Identifying Teaching Styles (IFITS) was developed to identify prevalent teaching styles (number of teaching styles utilized) by analyzing video-recorded lessons. During the video analysis, the rater indicates the instructor's teaching style every 20 s. At the end of the video analysis, the instrument reported the percentage of use for each teaching style during the lesson (30).

The System for Observing Fitness Instruction Time (SOFIT) is a tool that evaluates the amount and type of physical activity performed during the lesson. Information about the lesson content and the quality of the instructor's interaction with children is also provided. A rater evaluates the lesson recorded with a dedicated schedule, giving every 20 s information about the type of physical activity (laying, sitting, standing, moderate activity, vigorous activity) and the interaction of the instructors to promote physical education practice (interaction with inside lesson topic, interaction with outside lesson topic, no interaction). At the end of the evaluation, the percentages for each type of physical activity (sedentary and active behavior), and instructor interaction (inside lesson topic, outside lesson topic and absence of interaction) are provided for each variable analysed (31).

The Self-Control Questionnaire (SCQ) describes instructors’ self-estimated level of self-control. The questionnaire consists of 36 items, each rated on a five-point Likert scale, ranging from 1 (“not at all like me”) to 5 (“very much like me”) (32).

The Empathy Scale for Teachers (EST) is a self-evaluation questionnaire designed to measure the level of empathy among instructors. The questionnaire consists of 19 questions; each is rated on a five-point Likert scale, ranging from 1 (strongly disagree) to 5 (strongly agree) (33).

The Aquatic Perceived Competence Pictorial Scale (APCPS) is a perceptual scale that assesses a child's perception of swimming context/environment. As the APCPS is a pictorial scale, each increasing degree, illustrated by the images, corresponds to a score of 1–3. The final score ranges from 1 (low perception of aquatic ability) to 3 (high perception of aquatic ability). In addition, perceptions of children's autonomy, enjoyment, inclusion, and self-efficacy, were evaluated using specific questionnaire items. The final value of each score ranged from 1 to 3. The APCPS was administered to the children before a swimming lesson; they completed the written form of the scale by marking the figure that best reflected their experience (34, 35).

The Pictorial Scale of Perceived Water Competence (PSPWC) is a pictorial scale that allows for the assessment of children's actual aquatic abilities. Various exercises focused on basic aquatic skills are described and proposed for the children who perform them. The children's instructor must identify the image that best corresponds to the specific aquatic ability the child has reached among the given options. Each increasing degree illustrated by the images is associated with a score ranging from 1 (not able) to 3 (able). The values represent the final score, ranging from 1 (low level of water competence) to 3 (high level of water competence). This scale was used also for the aquatic perception competences of children. In this case PSPWC was administered by researchers (not involved in the teaching process) (36).

A retention test (RT) was administered four weeks after the posttest to evaluate the persistence of learning following a period without practice and to assess motor skill retention over time. Consistent with previous motor learning research, a minimum interval of 10 min was considered sufficient to assess short-term retention, whereas longer intervals ranging from days to months were used to evaluate long-term retention (37, 38). The retention test reflects the stability of motor memory after consolidation processes have occurred, allowing newly acquired skills to become relatively enduring over time. Consequently, retention performance indicates the extent to which learning has been maintained in the absence of practice. Moreover, retention outcomes may be influenced not only by the initial conditions under which learning occurred but also by the quality of the learning experience, including motivational and emotional factors known to enhance memory consolidation and long-term retention (39–41).

Semi-structured interviews were conducted to assess the transfer of swimming skills to environments outside the swimming pool (i.e., aquatic competence in open water). Parents of both experimental and control groups were interviewed using the following base questions: a) “Did you perceive from your child a transfer of competencies from the swimming pool to the natural environment or other swimming contexts different from the swimming pool?”; b) “Do you think your child can adapt and swim safely in an aquatic environment?”; c) “Did you perceive a change in your child's emotional and behavioural aspects when swimming in contexts different from the swimming pool?”; d) “Did you perceive any other changes in your child when approaching swimming contexts different from the swimming pool?”. The interviews were analysed by two different evaluators using the grounded theory methodology (42).

### 2.3 Experimental protocol design

**Figure 2:**
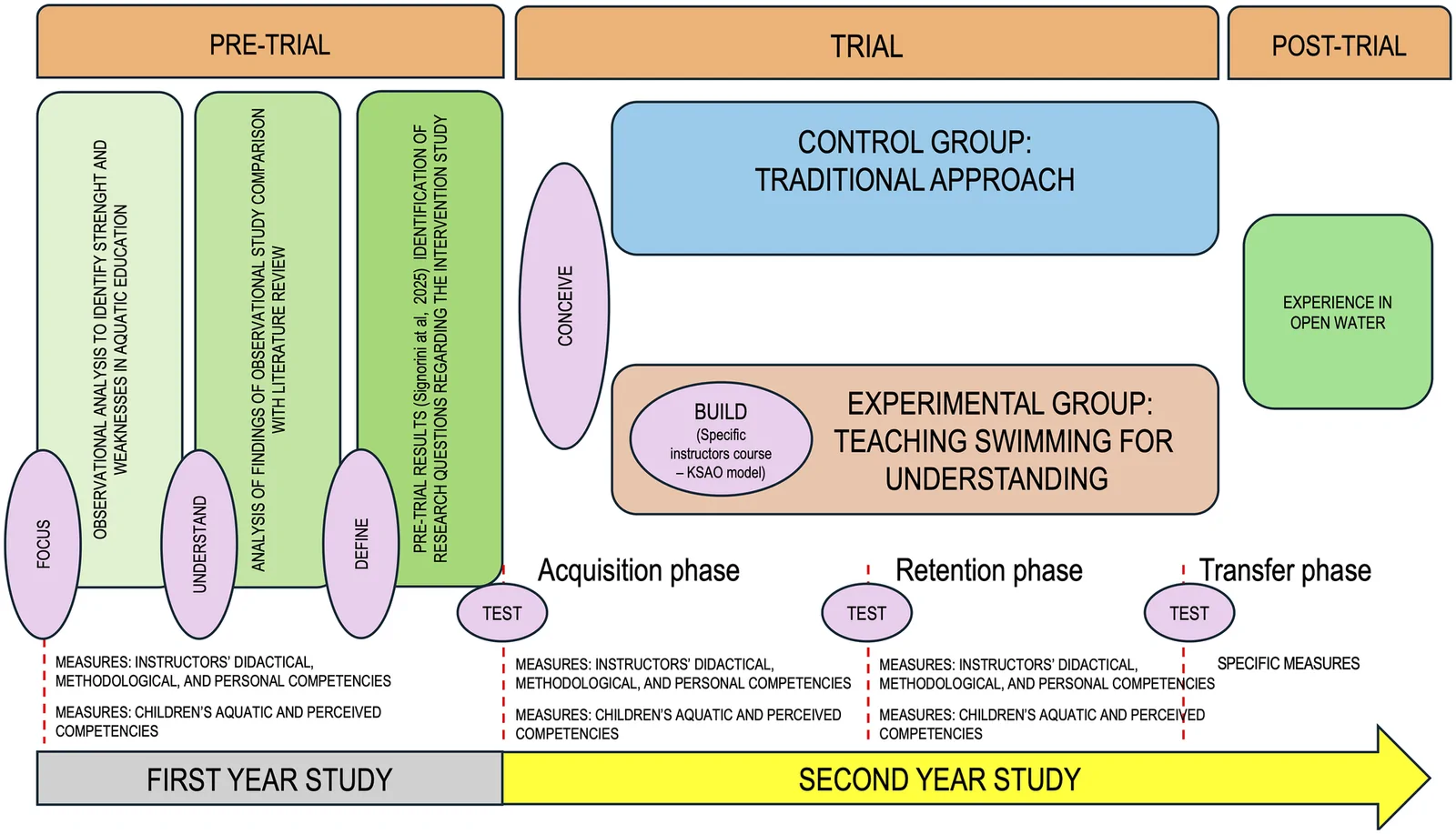
Timeline of the study.

The project employs an empirical, descriptive-observational research approach combined with experimental comparative research. These methodologies culminate in action research and a base research design. The process unfolds in several phases (Figure 2) (43).

- Focus: Observational analysis to identify the strengths and weaknesses in aquatic education.
- Understand: Literature review on contemporary learning and analysis of findings from the observational study.
- Define: Identification of research questions regarding the effectiveness of the TSfU approach compared to traditional methods.
- Conceive a Plan: Planning the training intervention for instructors.
- Build: Implementation and adaptation of the TSfU approach to the specific aquatic context.
- Test and Evaluation Tools: Periodic measurement of progress and adjustments to the intervention protocol.

A previous observation (3) confirmed the instructors’ predominant use of monoteaching pedagogy, primarily characterized by linear (command and practice) styles. The swimming instructors exhibited general positive personal skills (empathy and self-control). Moreover, instructors showed low interaction with children during practice and low promotion of physical activity outside the lesson. The involved children showed a discrepancy between actual and perceived aquatic competence, with the latter overestimating the former. In addition, children reported low levels of perceived autonomy and inclusion during swimming practice. As the present research project aimed to develop competencies (abilities and personal competencies) in swimming instructors, following the principles of the KSAO model, all previously mentioned aspects were considered weak points and main topics for the training of swimming instructors. These weak points and relative competencies were further assessed at the end of the intervention period. Supplementary Tables S2–S4 shows main characteristics of the intervention.

### 2.4 Statistical analysis

The normality of the data was assessed using the Shapiro–Wilk test. Differences between the TSfU and TS groups were evaluated at pre- and post-intervention and after a break from swimming lessons (retention). In particular, the 3 × 2 ANOVA analyzed the interaction Time (pre-post-retention)*Group (TSfU and TS) for children's actual and perceived aquatic competencies, differences between actual and perceived aquatic competencies, instructors’ methodological competencies, IESPES, IFITS, and SOFIT. The instructors’ personal competencies were evaluated using the same analysis, only focusing on pre-post results. The *post hoc* test was assessed using the Bonferroni correction. The effect size *η*^2^*_p_* was considered using the following cutoffs: *η*^2^*_p_* < 0.06 (small effect), 0.06 < *η*^2^*_p_* < 0.14 (medium effect), and *η*^2^*_p_* > 0.14 (large effect). To compare TSfU and TS in competence transfer, the Mann–Whitney *U* test was performed. The effect size was evaluated using Cohen's D with the following cutoffs: *d* = 0.20 (small), *d* = 0.50 (medium), *d* = 0.80 (large). The significance level was set at *p* < 0.05.

The qualitative analysis was used to interpret the final interview about the transfer of the swimming course. The analysis followed the grounded theory methodology and comprised a phase of open coding in which pre-labels were extrapolated from transcriptions of interviews with both the experimental and control groups, grouping the main themes that emerged. After this phase, similar themes were grouped into comprehensive labels. Successively, labels were also grouped into categories, quantified by how often they appeared in the interview. According to Corbin and Strauss (42), categories with a frequency below 50% were considered “sporadic,” those in the 51%–70% range “typical,” and those above 71% “general.” The category with the highest frequency was considered the core category. For each category, the percentages of the experimental and control groups were specified. To define the transfer effect, the following formula has been applied to the category percentages of experimental and control groups: Transfer % = [(*E* − *C*)/(*E* + *C*)] × 100 (37), where E represent the value of experimental group while C represent the value of control group. Two evaluators conducted the grounded theory process that was subsequently presented and discussed with the research team.

## 3 Results

### 3.1 Reliability

The IESPES obtained intra-rater ICC values of 0.993 and inter-rater ICC values of 0.982. IFITS and SOFIT evaluations yielded agreement values of 81% for intra-rater reliability and 87% for inter-rater reliability.

### 3.2 Swimming instructors

**Figure 3:**
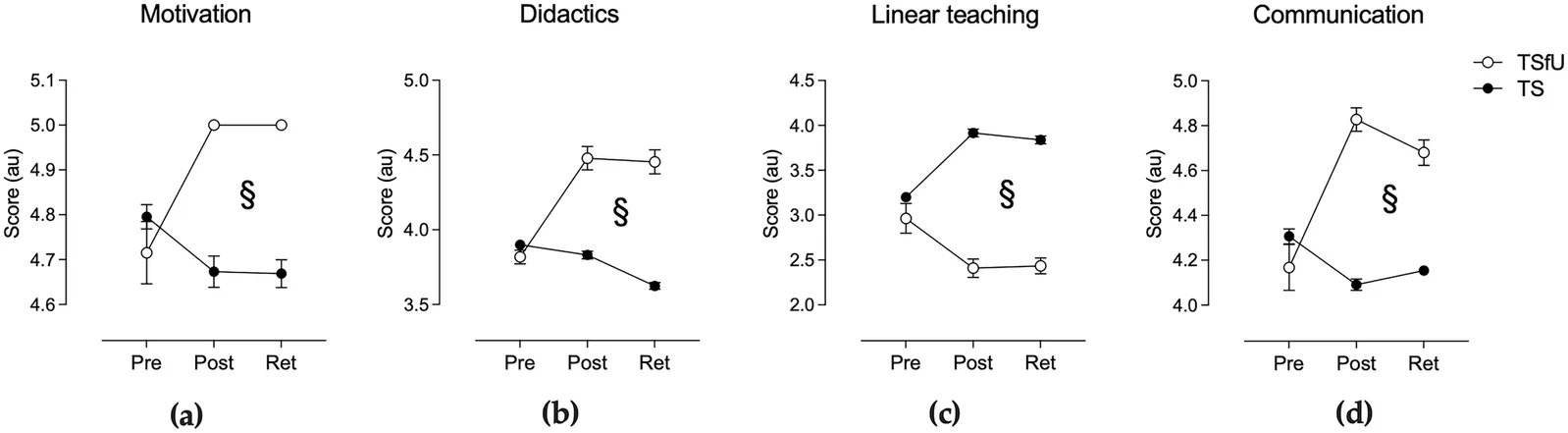
IESPES results. Panels display: **(a)** motivation, **(b)** didactics, **(c)** linear pedagogy, and (**d**) communication aspects in swimming lessons. §, significant interaction time*group (*p* < 0.05).

Results of IESPES (Figure 3) revealed a significant interaction time*group in motivation (*η*^2^*_p_* = 0.341, *p* < 0.001), didactics (*η*^2^*_p_* = 0.669, *p* < 0.001), linear teaching (*η*^2^*_p_* = 0.632, *p* < 0.001) and communication (*η*^2^*_p_* = 0.729, *p* < 0.001). In particular after the experimental period, TSfU group increased and retained levels of motivation (pre vs. post: *p* < 0.01, *d* = 1.15; post vs. retention: *p* = 1.00, *d* < 0.01) and didactics (pre vs. post: *p* < 0.01, *d* = 2.19; post vs. retention: *p* = 1.00, *d* = 0.07) while communication decreased in retention phase (pre vs. post: *p* < 0.01, *d* = 1.9; post vs. retention: *p* < 0.001, *d* = 0.43) and the level of linear teaching decreased in post intervention (pre vs. post: *p* < 0.01, *d* = 0.99; post vs. retention: *p* = 1.00, *d* = 0.04). Conversely, the TS group showed a decrease in motivation (pre vs. retention: *p* < 0.01, *d* = 0.78) and didactics (pre vs. retention: *p* < 0.01, *d* = 0.92), decreased their levels of communication (pre vs. retention: *p* < 0.01, *d* = 0.48), and increased linear teaching (pre vs. retention: *p* < 0.01, *d* = 1.17).

**Figure 4:**
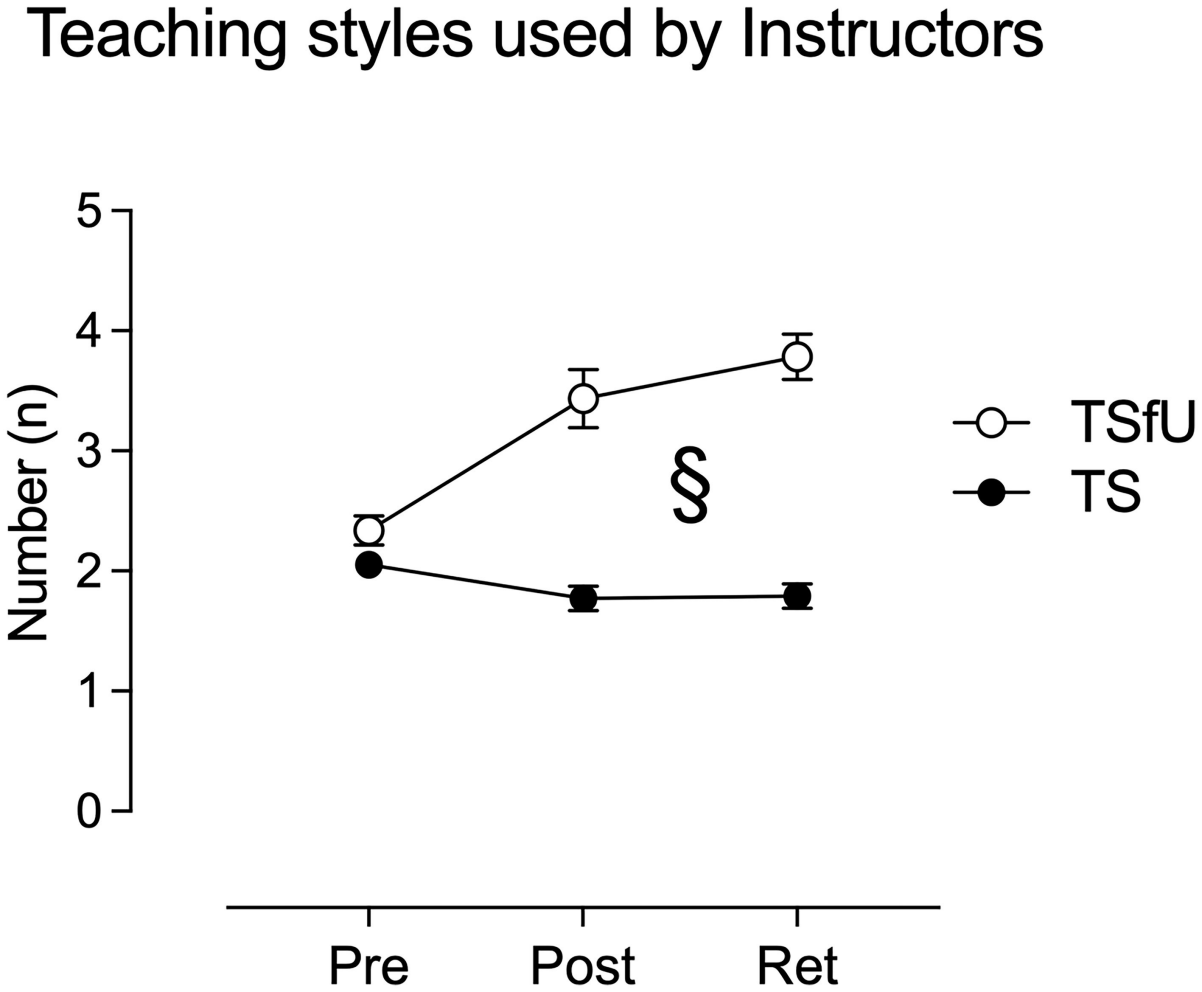
Number of teaching styles used in the pre- and post-intervention, and after the retention phase, measured by IFITS. Experimental, teaching swimming for understanding group (TSfU); Control, traditional swimming group (TS). §, significant interaction time*group (*p* < 0.05).

The IFITS analysis (Figure 4) revealed an interaction effect (*η*^2^*_p_* = 0.332, *p* < 0.001) also in the number of teaching styles used during swimming lessons. Indeed, the TSfU group increased the use of different teaching styles (pre vs. post: *p* < 0.01, *d* = 1.29), while the TS group maintained their usual practice (*p* = 0.65, *d* = 0.44).

**Figure 5:**
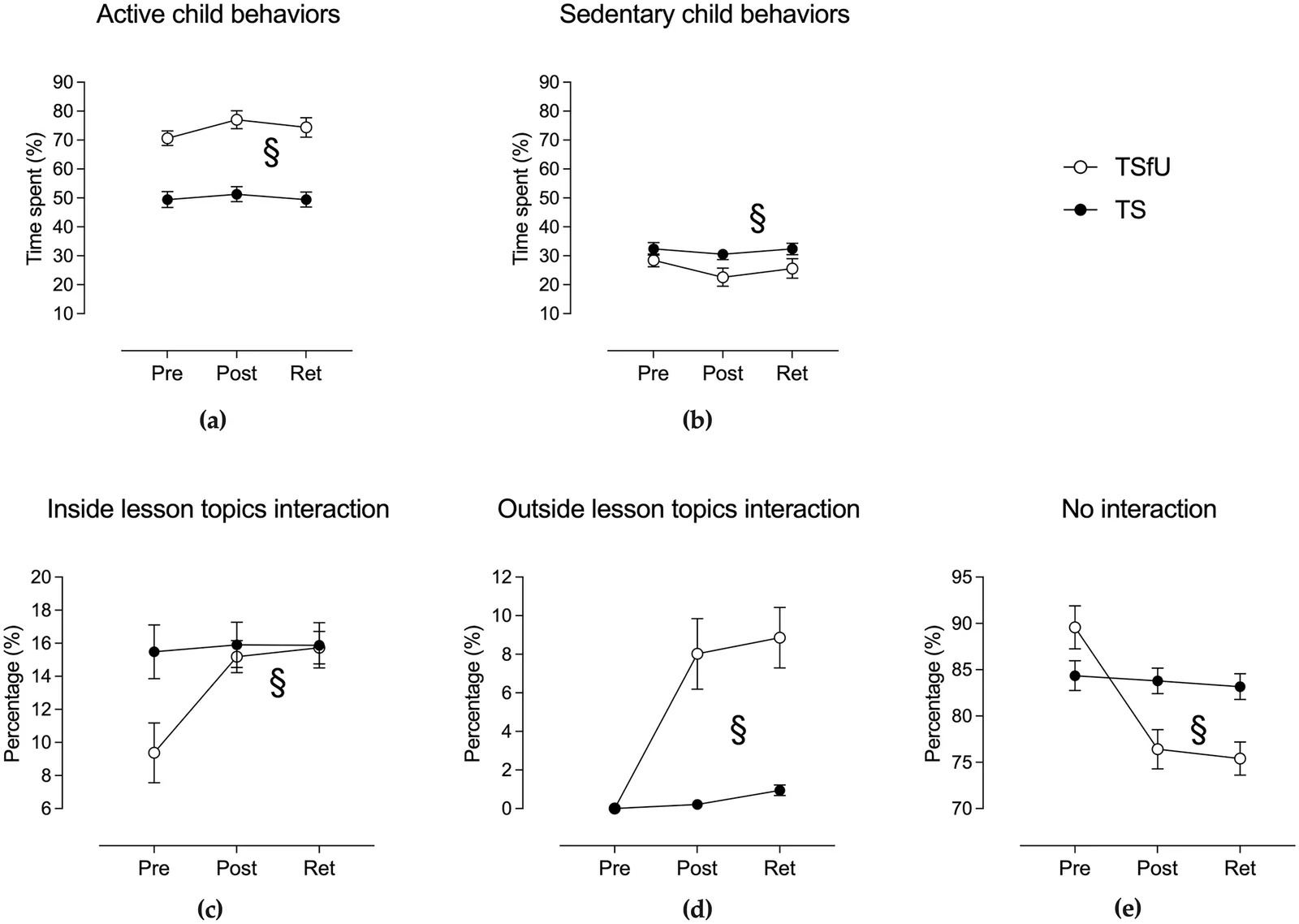
SOFIT (lesson intensity) analysis results. Panel **(a,b)** represent respectively active and sedentary behaviour during swimming lessons. §, significant interaction time*group. Experimental, teaching swimming for understanding group (TSfU); Control, traditional swimming group (TS). SOFIT (lesson interaction) analysis results. Panel **(c,d,e)** represent respectively interaction involving inside lesson topics, outside lesson topics and absence of interaction during swimming lessons. §, significant interaction time*group. Experimental, teaching swimming for understanding group (TSfU), Control, traditional swimming group (TS).

From the SOFIT analysis (Figure 5), it is evident that after the intervention period, the TSfU and TS groups responded differently (sedentary behavior: *η*^2^*_p_* = 0.027, *p* = 0.016; active behavior: *η*^2^*_p_* = 0.041, *p* = 0.002). TSfU group decreased the sedentary behavior (pre vs. post: *p* < 0.001, *d* = 0.38) and increased active behavior (pre vs. post: *p* < 0.001, *d* = 0.40) in post intervention but did not retain the results (active behaviors post vs. retention: *p* < 0.001, *d* = 0.17; sedentary behavior post vs. retention: *p* < 0.001, *d* = 0.19), while TS group maintained their level of intensity during swimming lessons (active behaviors pre vs. post: *p* = 0.464, *d* = 0.14; sedentary behavior pre vs. post: *p* = 0.644, *d* = 0.14).

Moreover, SOFIT revealed that instructors of TSfU increased their interaction levels (time*group interaction inside lesson topics: *η*^2^*_p_* = 0.198, *p* < 0.001; absence of interaction: *η*^2^*_p_* = 0.256, *p* < 0.001), also motivating through outside lesson topics (time*group interaction: *η*^2^*_p_* = 0.255, *p* < 0.001), while the TS group maintained their initial characteristics.

**Figure 6:**
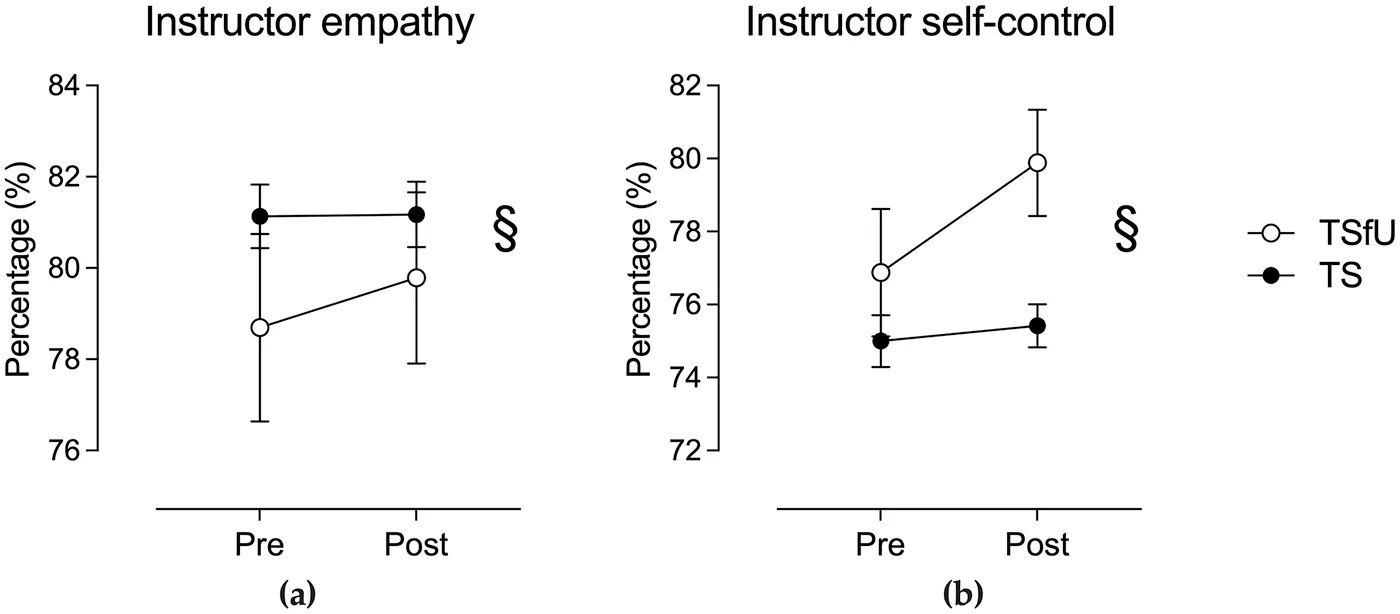
Instructors’ personal competences results. Panel a and b represent respectively instructors’ empathy and self-control. §, significant interaction time*group. Experimental, teaching swimming for understanding group (TSfU); Control, traditional swimming group (TS).

A similar trend was found in instructor's personal competencies results (Figure 6). Indeed, the intervention had positive effects on TSfU and maintained invariant TS group characteristics for both empathy (*η*^2^*_p_* = 0.103, *p* < 0.001) and self-control (*η*^2^*_p_* = 0.096, *p* < 0.001).

### 3.3 Children

**Figure 7:**
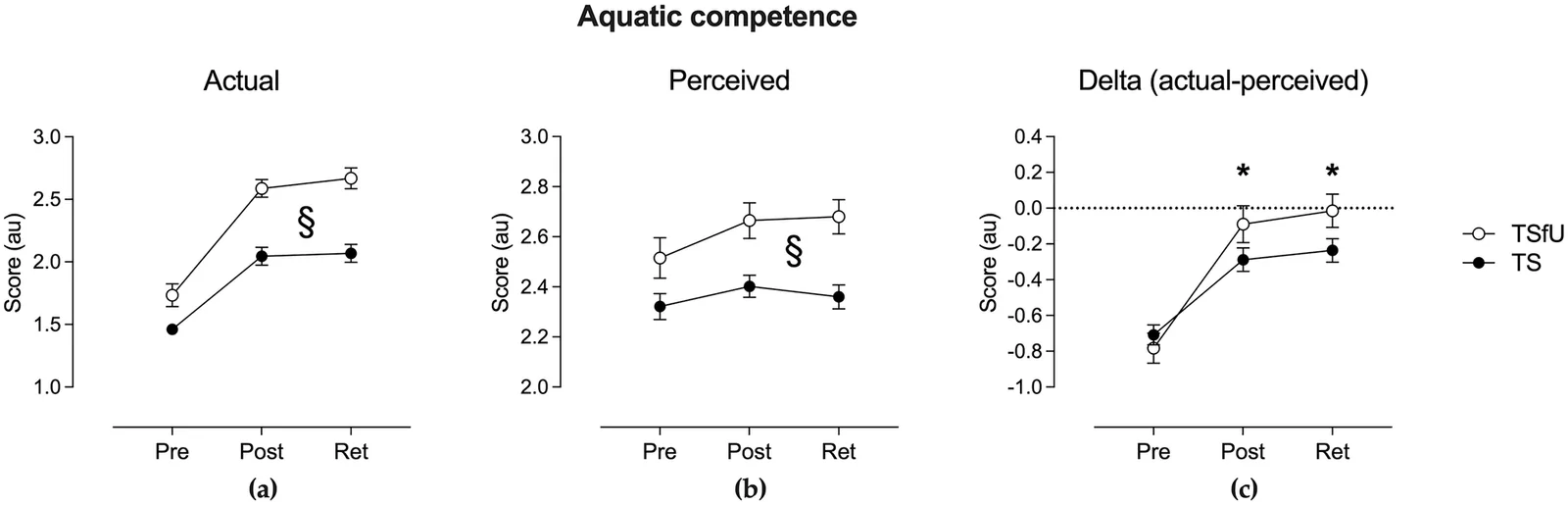
Children perceived and actual aquatic competence results. Panels represent respectively children's actual aquatic competence (panel a), children's perceived competence (panel b) and the differences between actual and perceived competence (panel c) in pre-post and retention phase. §, significant interaction time*group. *, significant difference experimental vs. control comparison (*p* < 0.05). Experimental, teaching swimming for understanding group (TSfU); Control, traditional swimming group (TS).

The analysis of the children's aquatic competences (Figure 7) revealed that the TSfU group increased their perceived (*η*^2^*_p_* = 0.023, *p* = 0.011) and actual competencies (*η*^2^*_p_* = 0.013, *p* < 0.001) more than the TS group, indicating a time*group interaction for both variables. In particular, TSfU increased both values post-intervention and maintained the level acquired during the retention phase, whereas the TS group showed similar trends but with a significantly smaller increase. Focusing on differences between actual and perceived aquatic competencies, no significant interaction was found (*η*^2^*_p_* = 0.013, *p* = 0.095); nevertheless, the *post-hoc* analysis revealed that TSfU showed significantly lower differences than the TS group in the post (*p* = 0.036, *d* = 0.48) and retention phases (*p* = 0.029, *d* = 0.47).

**Figure 8:**
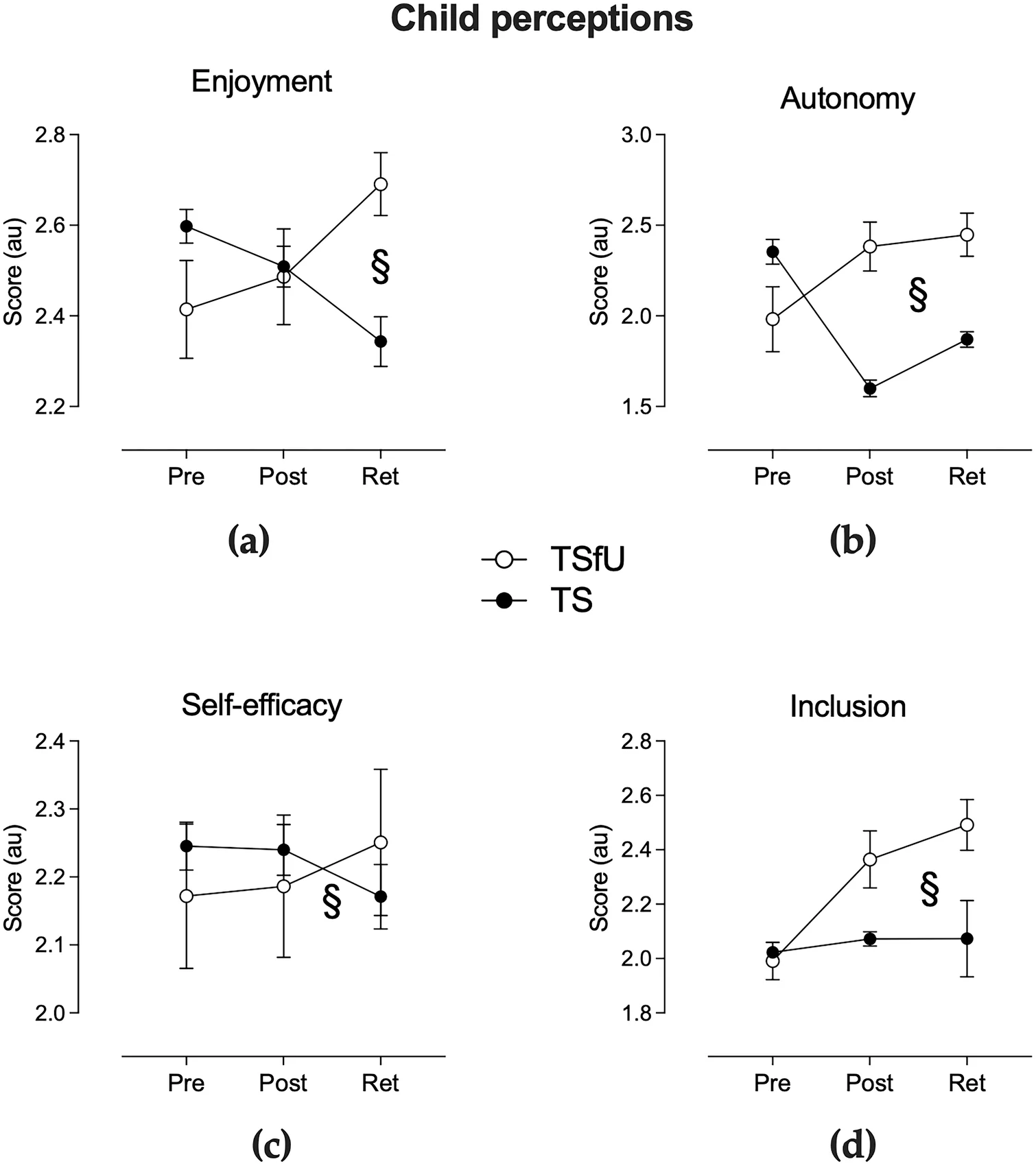
Children perceived aquatic competence subscales results. Panels represent respectively children's perceived enjoyment (panel a), children's perceived autonomy (panel b), self-efficacy (panel c), and perceived inclusion (panel d) during swimming practice in pre, post, and retention phase. §, significant interaction time*group. Experimental, teaching swimming for understanding group (TSfU); Control, traditional swimming group (TS).

Analyzing the subscales of the perceived environmental competencies (Figure 8), significant interaction time*group was found for perceived enjoyment (*η*^2^*_p_* = 0.312, *p* < 0.001), autonomy (*η*^2^*_p_* = 0.295, *p* < 0.001), self-efficacy (*η*^2^*_p_* = 0.064, *p* < 0.001), and inclusion (*η*^2^*_p_* = 0.249, *p* < 0.001). Enjoyment showed a positive trend, allowing the TSfU group to surpass the TS group after the retention period (Experimental vs. Control: *p* < 0.001, *d* = 0.720). Autonomy seemed to decrease in the TS group (pre vs. post: *p* < 0.001, *d* = 0.193; pre vs. retention: *p* < 0.001, *d* = 0.551) while increasing in TSfU in post-intervention (pre vs. post: *p* = 0.004, *d* = 0.152) and maintained in the retention phase (pre vs. post: *p* < 0.001, *d* = 0.584). TSfU and TS groups showed similar levels of self-efficacy. However, the TSfU group significantly increased their level (pre vs. retention: *p* = 0.05, *d* = 0.157), while the TS group maintained their status (pre vs. retention: *p* = 0.182, *d* = 0.149). Finally, the TS group maintained their levels of perceived inclusion (pre vs. retention: *p* = 0.444, *d* = 0.239), while the TSfU group increased (pre vs. retention: *p* < 0.001, *d* = 1.239).

### 3.4 Transfer of swimming teaching in natural environment

**Figure 9:**
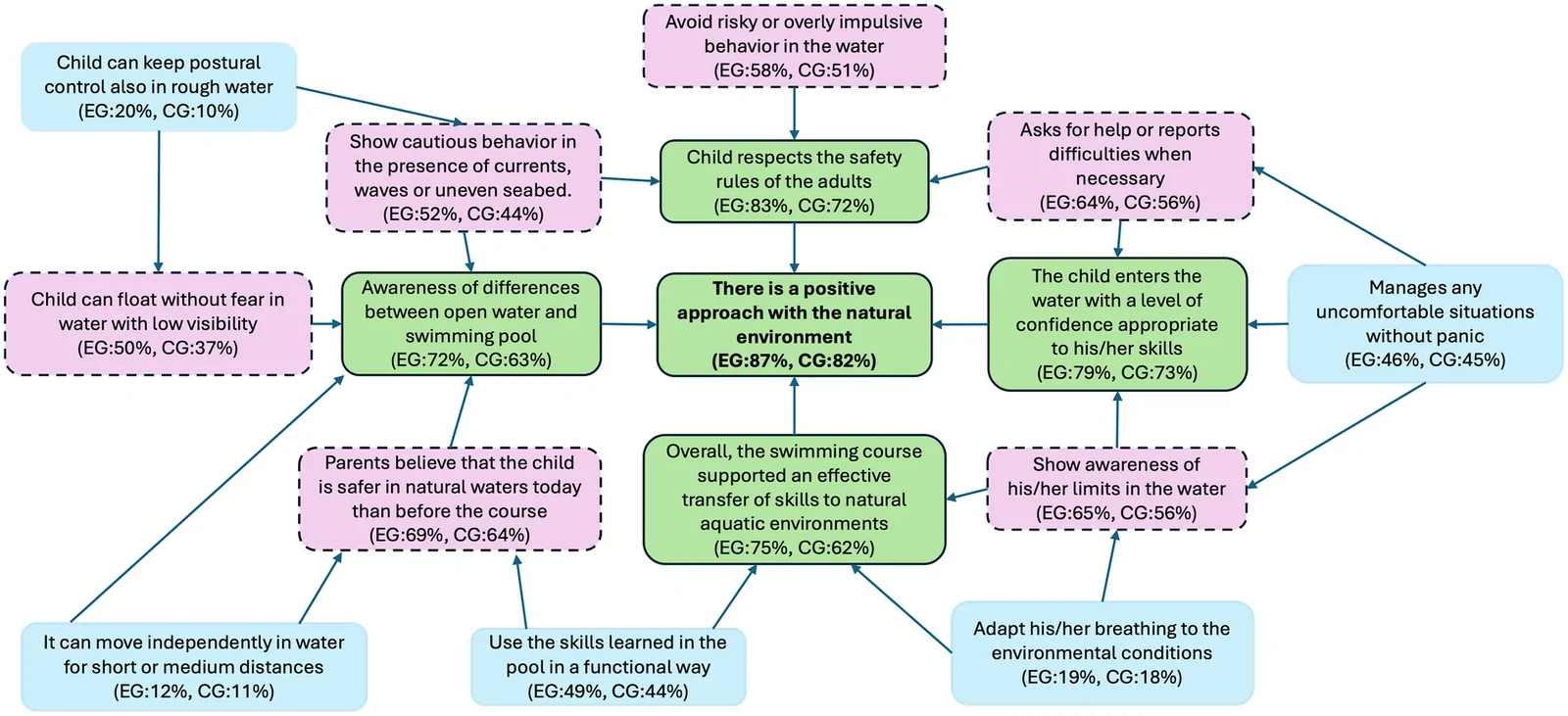
Transfer of swimming teaching results. Green boxes with continuous lines represent the general categories (recurred up to 71% of interviews), while the core category is evidenced in bold. Purple boxes with dash lines represents the typical categories (recurred from 50% to 70% of interviews) and the light blue boxes represent the sporadic categories (recurred less than 50% of the inter-views). In each box are presented percentages of experimental (EG) and control (CG) groups.

Interviews provide evidence of the intervention's positive impact on the experimental group. This impact is evidenced by the results of the transfer effect (Transfer % = [*E* − *C*)/(*E* + *C*)] × 100). Indeed, the interviews reported a mean transfer effect of 7.7% (in favor of the experimental group). The major effect is shown in category “Child can keep postural control also in rough water” (34.6%), and the lowest effect is shown in category “Manages any uncomfortable situations without panic” (0.9%). More specifically, the interviews reported that swimming course had positive effect on the approach to natural environment (transfer % = 3.3%), due to higher consciousness of children regarding his/her skills (transfer % = 3.8%), the respect of rules (transfer % = 7.0%), the awareness of difference between open water and swimming pool (transfer % = 6.3%). Moreover, parents reported a positive transfer effect from the swimming course (transfer % = 4.4%). Awareness of one's own skill development is reinforced by the ability to manage uncomfortable situations without panic or fear (transfer % = 0.9%), showing awareness of one's own limits (transfer % = 7.8%), and asking for help when necessary (transfer % = 6.5%). Furthermore, parents report that children tend to avoid impulsive behaviors in water (transfer % = 6.3%), showing caution in the presence of currents, waves, or an uneven seabed (transfer % = 8.2%). Finally, the children's swimming skills, such as floating (transfer % = 14.6%), postural control (transfer % = 34.6%), breathing (transfer % = 3.8%) and independence in water (transfer % = 6.4%) are perceived as functional (transfer % = 5.2%) also in a natural environment. Results of grounded theory are shown in Figure 9.

## 4 Discussion

This study examined whether a two-year Teaching Swimming for Understanding (TSfU) intervention was associated with changes in instructors’ pedagogical competencies and children's psychomotor, cognitive, and psychosocial outcomes compared with traditional technique-based swimming instruction. It also examined whether the children's outcomes were maintained following a one-month interruption and whether parents reported the transfer of aquatic competencies and behaviours to environments beyond the swimming pool.

Overall, the findings address the gap identified in the Introduction concerning the limited evidence available for sustained, understanding-oriented perspectives in aquatic education (2, 25, 26). Compared with traditional instruction, TSfU was associated with favourable changes at two interconnected levels. At the instructional level, instructors reduced their reliance on predominantly linear teaching, used a wider range of teaching styles, increased their interactions with children, and improved several didactic, communicative, relational, and self-regulatory competencies. At the learner level, children showed greater improvements in actual aquatic competence and generally more favourable patterns in enjoyment, autonomy, self-efficacy, and inclusion. Several of these outcomes were maintained following the interruption, while parents also reported modest but favourable evidence of transfer to natural aquatic environments.

These findings are consistent with the broader conception of aquatic competence presented in the Introduction. Aquatic education should not be limited to the reproduction of formal swimming strokes but should enable learners to perceive environmental information, regulate their actions, solve movement problems, evaluate risks, and adapt their behaviour to changing aquatic situations (4, 5). The present results suggest that TSfU may contribute to this broader educational objective by combining technical development with aquatic understanding, active decision-making, calibrated self-perception, and psychosocial participation.

The findings are also coherent with the theoretical transition from technique-dominated instruction to TGfU described by Bunker and Thorpe and subsequently developed through situated and understanding-oriented perspectives (6–8). In TSfU, technical skills are not excluded; rather, they are introduced as functional solutions to meaningful aquatic problems. The results should therefore not be interpreted as evidence against technical instruction itself, but as support for embedding technical instruction within a broader pedagogical process involving exploration, questioning, reflection, decision-making, and adaptation.

### 4.1 Changes in instructor competencies

The KSAO evaluation model was used in this study to organize and assess instructors’ knowledge, observable skills, broader abilities, and other professional characteristics (20, 21). It was not intended as a theory explaining children's motor learning. Within this evaluation model, the observed changes suggest that TSfU training affected not only what instructors knew about aquatic teaching, but also how they designed activities, communicated with learners, adapted tasks, and regulated their behaviour during instruction. Instructor motivation and didactic competence increased following the intervention and were largely maintained at retention. At the same time, reliance on linear teaching decreased and the number of teaching styles used increased. This pattern directly addresses one of the limitations of traditional swimming instruction identified in the Introduction: a predominantly linear structure may provide relatively few opportunities for learner autonomy, exploration, decision-making, and adaptation to individual needs (1–3). The results are also consistent with previous evidence showing that multiteaching and active-reflection approaches can broaden instructors’ methodological repertoires and increase learner participation (3, 44). From a TGfU perspective, the transition from monoteaching to multiteaching is important because meaningful problem solving requires instructors to move flexibly between questioning, guided discovery, task adaptation, feedback, observation, and, when required, explicit instruction (6–8). The decrease in linear teaching should therefore not be interpreted as the elimination of direct teaching. In aquatic environments, explicit guidance remains necessary for safety-critical competencies, for tasks carrying substantial consequences if performed incorrectly, and for learners requiring additional assistance. The pedagogical difference lies in avoiding the use of repetitive technical reproduction as the exclusive structure of learning.

The findings can also be interpreted through nonlinear pedagogy and ecological dynamics. These perspectives conceptualize learning as emerging from interactions among the learner, the task, and the environment (9–14). In this context, instructors are not simply transmitters of an ideal movement pattern. They design learning environments, manipulate constraints, observe individual responses, and guide learners toward the discovery of functional solutions. The increase in teaching-style variety is therefore consistent with the requirement to adapt instruction to differences in children's previous experience, confidence, physical characteristics, emotional responses, and emerging movement solutions. The findings from the video-analysis support this interpretation. TSfU instructors increased active lesson time and instructor–child interaction while reducing sedentary lesson time. Questioning, short periods of reflection, and individualized feedback may have directed children's attention toward relevant relationships among breathing, balance, propulsion, body orientation, and the effects of their actions in water. This is consistent with the constructivist and situated-learning foundations of TGfU (7, 8) and with nonlinear pedagogical principles emphasizing exploration and attention to relevant environmental information (9–14). An appropriately designed external focus of attention may also have contributed to the learning process when adapted to the task and the learner's developmental level (16).

IESPES Communication competence increased markedly from pre- to post-intervention. Although it decreased during the retention phase, it remained substantially above baseline. This pattern indicates partial attenuation of the maximum improvement rather than a return to initial levels. Communication behaviours such as questioning, individualized explanation, emotional reassurance, and responsive feedback depend on regular interaction with learners and may therefore require continuous practice and reinforcement.

The results concerning empathy and self-control extend this interpretation beyond methodological competence. Empathy showed a favourable, although relatively modest, change in the TSfU group, whereas self-control showed a clearer improvement compared with traditional instruction. These competencies may be particularly relevant in aquatic education, where children can differ substantially in confidence, fear, perceived difficulty, previous aquatic experience, and responses to uncertainty. Empathy may help instructors recognize these differences and adjust task difficulty or support without removing the challenge necessary for learning. Self-control may contribute to calm and consistent instructional decisions when children encounter fear, frustration, unexpected movement responses, or safety-related difficulties (45). These relational and self-regulatory competencies complement the didactic and communication components of the KSAO framework (20, 21). They are also consistent with the multidimensional conception of learning introduced earlier, according to which motor, cognitive, affective, and social outcomes are interdependent and influenced by the educational context (22–24). The parallel development of instructor and child outcomes is compatible with the proposed TSfU mechanism but should not be interpreted as proof of a specific causal pathway.

### 4.2 Children's aquatic and psychosocial outcomes

Actual aquatic competence increased in both groups, but the increase was greater in the TSfU group and was maintained following the interruption. This finding indicates that TSfU was associated with objectively assessed aquatic learning and not only with increased enjoyment or favourable self-perceptions. The result is consistent with the premise that aquatic competence extends beyond the reproduction of isolated swimming techniques (4, 5). Within TSfU, children experienced meaningful aquatic problems involving flotation, breathing, balance, body orientation, propulsion, support, direction, and environmental variability. Guided exploration and task modification may have helped them identify functional relationships between their actions and the behaviour of the aquatic environment. This interpretation is consistent with nonlinear pedagogy, in which movement solutions emerge through the manipulation of individual, task, and environmental constraints (9–14). Rather than prescribing an identical movement response for every learner, TSfU provided opportunities to explore different solutions while instructors retained responsibility for defining objectives and maintaining safety. Previous research on nonlinear approaches in young swimmers similarly suggests that representative and variable learning experiences may support functional skill acquisition (46). The present study extends this literature by showing favourable outcomes following a sustained intervention involving both instructor development and child learning.

The results also support the distinction between swimming skill and broader aquatic competence. Technique remains an important component, but competence requires learners to integrate perception, movement, decision-making, and adaptation (4, 5). The greater improvement observed in the TSfU group may therefore reflect not only additional technical proficiency but also the development of more functional relationships among movement, task requirements, and environmental information. However, because the objective assessment was conducted in the swimming-pool context, the results should not be interpreted as demonstrating competence in every aquatic environment.

Perceived aquatic competence and actual aquatic competence represent related but distinct constructs. Perceived aquatic competence concerns children's judgments about their ability to perform specific aquatic actions, whereas actual competence concerns objectively assessed performance (34, 36). At baseline, perceived competence was higher than actual competence. During the intervention, perceived competence changed less markedly than actual competence, and the discrepancy between the two measures progressively decreased. At retention, the measures were almost aligned in the TSfU group. This improved correspondence may be important from a water-safety perspective. Safe behaviour requires both sufficient competence and a realistic judgment of whether that competence is adequate for the demands of a particular situation (5). Overestimation may encourage risk-taking, whereas substantial underestimation may restrict participation and learning. The results therefore suggest that TSfU may support not only aquatic performance but also more calibrated self-perception. Nevertheless, this calibration was demonstrated only for the competencies and conditions assessed in the study and cannot be generalized automatically to unfamiliar open-water situations.

The psychosocial findings further support the multidimensional approach to aquatic education (1, 22–24). Enjoyment, autonomy, self-efficacy, and inclusion showed generally more favourable patterns in the TSfU group, although their magnitude and temporal development differed. Enjoyment increased, particularly at retention, whereas it decreased in the traditional group. Autonomy increased following TSfU and remained elevated, while the traditional group showed a marked reduction. Inclusion also increased and remained high in the TSfU group. Self-efficacy showed a more moderate improvement but contributed to the overall favourable psychosocial profile. These findings are consistent with self-determination theory, according to which learning environments supporting competence, autonomy, and meaningful participation may promote intrinsic motivation and engagement (17, 18). TSfU provided children with opportunities to make meaningful choices, explore solutions, receive individualized support, and experience progressively achievable challenges. These features may have supported children's enjoyment and active involvement.

The findings are also relevant to aquatic physical literacy. Physical literacy includes not only movement competence but also motivation, confidence, knowledge, understanding, and willingness to participate in physical activity throughout life (19, 22). Previous aquatic-literacy research has similarly emphasized the value of pedagogical approaches that integrate perceived and actual competence rather than focusing exclusively on technical execution (1, 2). By producing parallel improvements in aquatic and psychosocial outcomes, TSfU appears compatible with this broader educational perspective.

Autonomy within TSfU should not be equated with the absence of instructional structure. Instructors continued to establish objectives, manipulate task conditions, monitor safety, and provide explicit guidance when necessary. Children nevertheless assumed a more active role in recognizing aquatic problems, exploring possible responses, and evaluating the effects of their actions. Thus, the findings support a form of structured autonomy rather than unrestricted discovery (47). The increase in inclusion may similarly be related to task adaptability. By modifying interaction, support, depth, distance, orientation, object use, and task difficulty, instructors could maintain a common educational objective while enabling children with different initial competencies to participate meaningfully. This interpretation is consistent with literature describing aquatic literacy and cooperative or multidimensional learning as involving simultaneous motor, cognitive, affective, and social outcomes (2, 22–24).

### 4.3 Retention of child outcomes

The maintenance of several child outcomes following the one-month interruption suggests that the intervention produced changes extending beyond immediate performance. Actual aquatic competence was maintained and showed a further small increase in the TSfU group, while perceived competence remained comparatively stable. The correspondence between objectively assessed and perceived competence consequently improved. These results are consistent with the acquisition–retention–transfer framework introduced earlier in the manuscript. Acquisition refers to the development of functional competencies during practice, whereas retention concerns the availability of those competencies following a period without structured practice. The maintenance of actual aquatic competence therefore provides stronger evidence of learning than an immediate post-intervention improvement alone. From a nonlinear-pedagogy perspective, meaningful and variable practice may support retention because learners are required to reconstruct functional solutions rather than depend exclusively on the repeated reproduction of a prescribed movement pattern (9–14, 38). Within TSfU, children were encouraged to understand the aquatic problem, explore possible actions, and relate successful movement responses to relevant environmental information. This structure may have facilitated the stabilization of adaptable aquatic competencies. Meaningful participation, enjoyment, perceived competence, intrinsic motivation, and physical literacy may also contribute to continued engagement and the maintenance of learning (17–19). The fact that enjoyment, autonomy, self-efficacy, and inclusion were maintained or increased following the interruption is consistent with this interpretation.

Retention also differed across outcomes. This is not unexpected because actual competence, perceived aquatic competence, enjoyment, autonomy, self-efficacy, and inclusion represent distinct processes. The 1-month assessment provides evidence of short-term maintenance, but longer follow-up periods are required before conclusions can be drawn about durable learning across successive swimming courses or developmental stages.

### 4.4 Transfer beyond the swimming pool

Parents reported a mean transfer effect favouring the TSfU group, although the magnitude varied substantially across categories. The largest reported effect concerned maintaining postural control in rough water, whereas the difference regarding the management of uncomfortable situations without panic was small. Parents also described favourable patterns in awareness of personal limits, respect for safety rules, caution in the presence of waves or currents, help-seeking, floating, breathing, and independence in natural aquatic environments.

These findings are consistent with the ecological-dynamics approach to water competence (4). From this perspective, transfer does not involve reproducing an identical movement in a different setting. It involves adapting behaviour to the information and action possibilities available in the new environment. The ability to float, control posture, regulate breathing, or select an appropriate action may change according to depth, stability, currents, waves, visibility, temperature, clothing, emotional pressure, and available support.

Representative learning design offers an additional explanation (15). Transfer is more likely when learning tasks preserve relevant perceptual information and action possibilities from the environments in which competencies will subsequently be used. TSfU activities involving variations in support, body orientation, depth, direction, objects, and water stability may have exposed children to a broader range of functional movement problems than stable and repetitive technique-based practice. The findings therefore provide preliminary support for the proposition that variable and meaningful aquatic practice can facilitate adaptation beyond the original instructional context (4, 15). They are also consistent with the broader definition of water competence, which includes safe judgment, risk awareness, behavioural adaptation, and emotional regulation in addition to formal swimming ability (5). Nevertheless, the transfer findings require cautious interpretation. They were based on parental reports and did not involve direct observation of children during unexpected events in open water. Parents’ perceptions may reflect actual behavioural changes, but they may also be influenced by expectations, previous experiences, or knowledge of the child's participation in swimming lessons. Furthermore, natural aquatic settings introduce demands that cannot be reproduced fully in a conventional swimming pool. Future assessments should distinguish among knowledge of an appropriate response, confidence, perceived competence, objectively assessed pool competence, performance under representative conditions, and actual behaviour during an unexpected situation. Carefully controlled assessments reproducing relevant environmental demands may provide stronger evidence while preserving participant safety (48).

### 4.5 Integrated interpretation and practical implications

Taken together, the findings suggest that TSfU modified the instructional environment in the direction anticipated by the theoretical framework presented in the Introduction. Instructors moved from predominantly linear and prescriptive teaching toward a more varied approach. These changes occurred alongside improvements in children's actual aquatic competence and generally favourable psychosocial outcomes. This dual-level pattern is important because previous literature has often examined either pedagogical practices or child outcomes, while evidence concerning sustained interventions addressing both instructors and learners remains limited (2, 3, 25, 26). The present study therefore contributes to the literature by examining how instructor professional development and children's aquatic learning may develop in parallel within a two-year intervention.

The findings also support an integrated interpretation of TGfU, nonlinear pedagogy, and aquatic physical literacy. TGfU provides the understanding-oriented structure in which learners engage with meaningful problems before or alongside technical refinement (6–8). Nonlinear pedagogy explains how functional movement solutions may emerge through interactions among the learner, task, and environment (9–14). Representative learning design provides a basis for creating activities that may support transfer (15), while physical-literacy and self-determination perspectives emphasize motivation, perceived competence, autonomy, and sustained participation (17–19, 22). The TSfU framework brings these elements together in relation to the distinctive motor, emotional, environmental, and safety requirements of aquatic education.

The practical implication is not that aquatic instructors should abandon technical teaching. Instead, technical instruction should be combined with meaningful aquatic problems and adapted to the learner's needs and to the safety requirements of the task. Direct instruction is particularly important for entries, jumps, unexpected falls, rescue-related actions, and other competencies in which an inappropriate response could create substantial risk. Guided discovery and questioning can then be used before or after explicit instruction to promote understanding of why a particular response is safe and functional. Instructor education is therefore central to implementation of methodological strategies. Effective TSfU delivery requires instructors to identify relevant aquatic problems, manipulate constraints purposefully, observe individual responses, provide informative feedback, use questioning appropriately, and balance autonomy with safety. The partial attenuation of communication competence during retention indicates that these skills may require continued professional support. Periodic mentoring, peer observation, reflective meetings, and booster training could help maintain the interactive and relational competencies developed during the intervention.

### 4.6 Limitations

A first limitation is the lack of a direct measure of the transfer, which was investigated by interviewing the children's parents. Indeed, the present research investigated aquatic competence in a swimming pool but did not examine it under specific observations in open water. Furthermore, transfer to everyday-life contexts (e.g., inclusion and autonomy in social life within a proactive transfer design framework) was not examined. A second limitation concerns long-term retention and consolidation. Retention was assessed after one month of rest with a limited number of trials. It would be valuable to evaluate retention over a longer period and across more trials to determine whether the skills acquired in the previous course are maintained at the start of a more advanced course. If learning is effectively consolidated, the acquired skills could be leveraged to increase physical activity levels, thereby improving health.

### 4.7 Future research perspectives and practical application

In summary, this study has examined the main aspects of the topic, highlighting the theoretical and practical implications of the results obtained. The data collected and the analyses conducted suggest important directions for future research, offering insights for further exploration in this area. The findings contribute to a deeper understanding of the phenomenon and can guide both practical and theoretical interventions. It is hoped that this work will stimulate further investigations and applications in the field.

**Figure 10:**
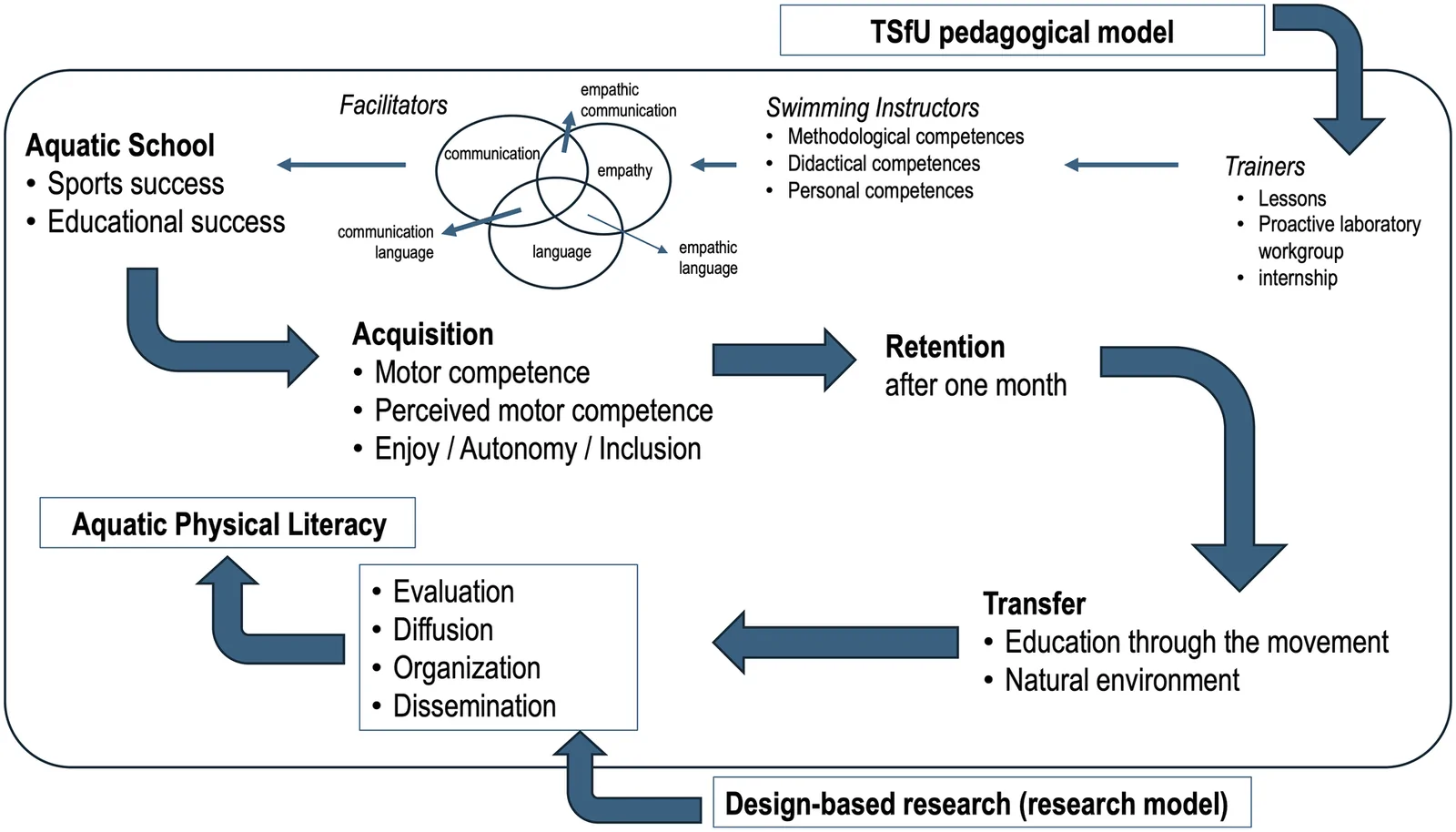
Summary of the training and research rationale.

Figure 10 summarizes the entire process characterizing an approach to educational research based on design-based research.

## 5 Conclusions

In conclusion, the results of the TSfU program demonstrate that the proposed aquatic education framework effectively promotes not only motor skill acquisition but also cognitive and socio-emotional growth in children. The findings suggest that personalized teaching strategies (a multi-teaching approach), which account for students’ diverse learning needs, are essential for enhancing both the effectiveness and sustainability of learning outcomes. By fostering a dynamic and interactive learning environment, TSfU has proven to be a valuable model for teaching swimming, aligning with both contemporary educational theories and the broader goals of lifelong learning and global well-being.

## Data Availability

The datasets presented in this study can be found in online repositories. The names of the repository/repositories and accession number(s) can be found below: https://zenodo.org/records/20715921.
