# Supplementary material for: The teaching swimming for understanding framework: an integrated approach to aquatic education for children and instructors
Source: Front Sports Act Living. 2026 Aug 12;8:1916430. doi: 10.3389/fspor.2026.1916430 (PMC13506936; doi:10.3389/fspor.2026.1916430)
Supplement: Supplementary file 1 [file Table1.docx]

Supplementary material

**Table S1.** Explanation of the KSAO model.

| **Features** | **Explanation** |
| --- | --- |
| Knowledge | Instructors acquire a strong theoretical foundation for effective teaching and an understanding of the psychological and emotional aspects of children's learning. Children, on the other hand, learn the basics of aquatic motor competencies and water safety, as well as the importance of understanding their limits and how to overcome challenges. |
| Skills | For instructors, these skills involve communication, motivation, and organizing lessons so that all children can actively participate. Children develop motor skills related to aquatic motor competencies (such as floating, kicking, and breathing), as well as social skills such as working in a group. |
| Abilities  (competencies) | Instructors must be able to read children's emotional and motor needs and continually adapt their teaching to keep them motivated and engaged. For children, the constructivist and ecological dynamics approach allows them to learn through practical experiences, building self-confidence as they acquire aquatic skills. |
| Other | Instructors must have strong motivation and passion for teaching, creating an emotionally safe and inclusive environment. Children, in the context of aquatic education, gain more autonomy and joy as they develop a sense of competence (self-efficacy), learning to be, and learning to be with others. |
| Learning process | The educational approach used and realized with the instructors to develop aquatic competencies in children. |
| Learning product | The effects of the educational approach evaluated through research outcomes. |
| Learning training | Based on frontal lessons, workshops with debates, group work, and internships, it has fostered the development of competent instructors in educational, methodological, and psycho-relational areas. In particular, the facilitators on whom attention was focused to ensure effective teaching to children included: empathetic communication, empathetic language, and appropriate verbal, non-verbal, and paraverbal communication. |

The table shows how the KSAO model can be applied to both instructors in training and children in acquiring aquatic and social skills. For instructors, the TSfU approach helps develop the pedagogical, communication, and motivational skills necessary to create a dynamic learning environment. For children, aquatic education not only fosters motor skill acquisition but also promotes autonomy, positive self-perception, and socialization, all essential elements of their psychological and emotional development.

**Table S2.** Application of the TSfU model.

| **Instructors (TSfU Training)** | **Children (Aquatic Education)** | **KSAO** |
| --- | --- | --- |
| - Knowledge of learning theories in particular ecological dynamics learning and constructivist pedagogy.  - Knowledge of aquatic motor competencies and water safety.  - Understanding of emotional and social dynamics in learning. | - Knowledge about the possibilities of the body in an aquatic environment.  - Familiarity with basic motor skills (floating, kicking, breathing) (procedural knowledge).  - Awareness of the importance of water safety. | Knowledge  (what I know)  Learning process |
| - Communication skills to adapt teaching to different ability levels and needs.  - Capability to organize activities that promote inclusivity and motivation.  - Capability to encourage participation and engagement. | - Development of motor skills for swimming and aquatic competence.  - Problem-solving capability related to aquatic tasks (e.g., breath control, coordination).  - Capability to work in groups and interact with others. | Skills  (what I can do)  Learning process |
| - Methodological competencies  - Teaching and communication competencies  - Personal competencies | - Water-related motor competencies  - Perception of water motor competencies  - Perception of psychosocial competencies | Abilities  (how much I can apply)  Learning product |
| - Motivation and empathy to build positive relationships with children.  - Interest and passion for teaching aquatic motor competencies and children's well-being.  - Resilience in managing group challenges. | - Autonomy and independence gained during learning, with increased self-efficacy (confidence in one's abilities).  - Inclusivity and emotional well-being (self-regulation and socialization).  - Joy in achieving motor and social goals. | Other  (how I am)  Learning product |

**Table S3.** The Acquisition, Retention, and Transfer (ART) process characterizing the approach to educational research based on design-based research.

| **The aquatic school** | | |
| --- | --- | --- |
| Following the learning approach model in line with the national and international goals and norms, represents a training concept focused not only on sports success but also on educational development. | | |
| ***Acquisition*** | ***Retention*** | ***Transfer*** |
| the construction of aquatic motor competence, perceived aquatic motor competence, enjoyment, autonomy, and inclusion. | the ability to retain these skills even after a period of inactivity (1 month), indicating their memorization. | the application of these skills in natural aquatic environments, as evidenced by the results of the interview administered to parents. |
| The key elements of this approach follow a road map characteristic of design-based research. Positive evaluation of these elements supports a methodological approach grounded in scientific research that can be replicated, organized, and disseminated across contexts, with the aim of promoting an increasingly profound aquatic culture. | | |

**Table S4.** Example of intervention contents.

| **Specifics** | **TS (traditional swimming group)** | **TSfU (Teaching swimming for understanding group)** |
| --- | --- | --- |
| Approach | Linear | Nonlinear |
| Criteria | - Simple/complex - Easy/difficult - Specific objectives | - Variation on constraints (organism, environment, task) - Affordances: integration of multiple psycho-pedagogical curvatures (motor, cognitive, affective, social) - Perception/action - Representative design - Multiteaching approach personalization |
| Contents | 1. First contacts with the water 2. Water on the face 3. Head in the water 4. Eyes open underwater 5. Breath control 6. First attempts at floating 7. First propulsions in water 8. Jumps, falls, dives in the water | 1. Environmental constraints  - Using a stable support for translocation (pool edge) - Using a stable and an instable support (pool edge and cable) - Using instable supports (two cables) - Using an instable support (a cable)   Special features: maintaining the perception/action ratio; Use of highly developed hands to pull oneself forward   2. Use of metaphors to identify the movements done by the children (shrimp, crabs, cuttlefish, at night) 3. Navigating under moving obstacles that each person chooses how to navigate ("monkey" lifting completely, the "crocodile" lifting partially, the "hippopotamus" without lifting)   Special features: affordances and attunement   4. Story telling   The teacher tells a story, and each child suggests how it might continue: oral language   5. Body scheme   “Swimming to the hospital”: children move freely while holding the part of the body indicated by the instructor with one hand   6. Temporality   Following a rhythm during translation (drawing on a paper or board or auditory stimulus)  OOOOO O O O O OO O OO O  Special features: applying to translation with cables   7. Spatiality   Creating geometric shapes using available equipment (boards, pull buoys, sticks)  Special features: assessment of reasoning skills, completing the task in groups as well (cooperative learning)   8. Communication   In various groups, the teacher encourages the children to build a castle or tower using the available materials, then asks, "How did you feel working with your mates?"  Special features: let the children choose their groupmates   9. Autonomy   Changing clothes and organizing personal belongings in the closet according to established guidelines  Special features: prescriptive approach, safety, order   10. Self-efficacy   When proposing activities to children, it is helpful to ask them whether they feel capable of carrying out the task and then encourage them to directly verify whether they were successful. This process helps them develop a greater awareness of the difference between the perception of their own abilities and their actual capacity to apply them, while observing over time how their sense of competence progressively improves.  Special features: assessing the awareness of own abilities   11. Conflict solving   During critical periods, contact with the pool or water  (e.g., entering the pool, submerging the head, jumping, and falling into the water). The instructor will promote reflection on conflict resolution through targeted questioning prior to providing explanations, thereby fostering a more participatory pedagogical approach. Metaphors, imaginative games, and storytelling will also be employed to facilitate engagement.   12. Transferability   Immediate exercises in shallow and deep water, constructive scenarios involving situational variability (e.g., moving around while wearing a T-shirt, creating waves and splashes—finding adaptive solutions)  Special features: creation of a representative design |
